# Unraveling Down Syndrome: From Genetic Anomaly to Artificial Intelligence-Enhanced Diagnosis

**DOI:** 10.3390/biomedicines11123284

**Published:** 2023-12-12

**Authors:** Aabid Mustafa Koul, Faisel Ahmad, Abida Bhat, Qurat-ul Aein, Ajaz Ahmad, Aijaz Ahmad Reshi, Rauf-ur-Rashid Kaul

**Affiliations:** 1 Department of Immunology and Molecular Medicine, Sher-i-Kashmir Institute of Medical Sciences, Srinagar 190006, India; 2 Department of Zoology, Central University of Kashmir, Ganderbal, Srinagar 190004, India; 3 Advanced Centre for Human Genetics, Sher-i-Kashmir Institute of Medical Sciences, Srinagar 190011, India; 4 Department of Human Genetics, Guru Nanak Dev University, Amritsar 143005, Punjab, India; quratain417@gmail.com; 5 Departments of Clinical Pharmacy, College of Pharmacy, King Saud University, Riyadh 11451, Saudi Arabia; 6 Department of Computer Science, College of Computer Science and Engineering, Taibah University, Madinah 42353, Saudi Arabia; aijazonnet@gmail.com; 7 Department of Community Medicine, Sher-i-Kashmir Institute of Medical Sciences, Srinagar 190006, India

**Keywords:** Down syndrome, neurodevelopment, cognitive impairment, comorbidity, diagnosis, management, artificial intelligence, machine learning, neurological disorders, intellectual disability

## Abstract

Down syndrome arises from chromosomal non-disjunction during gametogenesis, resulting in an additional chromosome. This anomaly presents with intellectual impairment, growth limitations, and distinct facial features. Positive correlation exists between maternal age, particularly in advanced cases, and the global annual incidence is over 200,000 cases. Early interventions, including first and second-trimester screenings, have improved DS diagnosis and care. The manifestations of Down syndrome result from complex interactions between genetic factors linked to various health concerns. To explore recent advancements in Down syndrome research, we focus on the integration of artificial intelligence (AI) and machine learning (ML) technologies for improved diagnosis and management. Recent developments leverage AI and ML algorithms to detect subtle Down syndrome indicators across various data sources, including biological markers, facial traits, and medical images. These technologies offer potential enhancements in accuracy, particularly in cases complicated by cognitive impairments. Integration of AI and ML in Down syndrome diagnosis signifies a significant advancement in medical science. These tools hold promise for early detection, personalized treatment, and a deeper comprehension of the complex interplay between genetics and environmental factors. This review provides a comprehensive overview of neurodevelopmental and cognitive profiles, comorbidities, diagnosis, and management within the Down syndrome context. The utilization of AI and ML represents a transformative step toward enhancing early identification and tailored interventions for individuals with Down syndrome, ultimately improving their quality of life.

## 1. Introduction

Down syndrome, first described by John Langdon Down in 1866, is a genetic disorder characterized by the presence of an additional chromosome 21 due to non-disjuntioning during gametogenesis and is reportedly the most common chromosomal abnormality in humans [1,2,3,4]. Down syndrome is a genetic disorder characterized by the presence of an extra copy of chromosome 21. It manifests in three main types: trisomy 21, translocation Down syndrome, and mosaicism [5]. Trisomy 21, accounting for the majority of cases, involves an extra copy of chromosome 21 in every cell [6]. Translocation Down syndrome occurs when the extra copy is attached to another chromosome [7]. Mosaicism, the least common type, involves a mixture of cells with two and three copies of chromosome 21 [7,8,9]. Patients suffering with this disorder show mild to moderate intellectual disability, retarded growth besides other peculiar facial features [10].

The incidence of Down syndrome, ranging from 1 in 319 to 1 in 1000 live births, escalates with advanced maternal age, surpassing 200,000 cases annually globally [11,12]. It is established in the scientific literature that the occurrence of other autosomal trisomy is much more common than trisomy 21 but owing to their poor postnatal survival, Down syndrome takes a lead in being the most frequently occurring live born aneuploidy (trisomy 21) [13]. Differences in the incidence and presentation of Down syndrome based upon the ethnic and geographic background are also reported [14]. Besides the occurrence of non-disjunction in chromosome 21 during gametogenesis, there are other factors that can lead to trisomy 21, including Robertsonian translocation, isochromosome formation, and the presence of a ring chromosome [6]. Isochromosome formation entails the simultaneous separation of two long arms, as opposed to one long and one short arm, and this phenomenon is observed in approximately 2% to 4% of patients [15,16]. In cases of Robertsonian translocation, the long arm of chromosome 21 becomes fused with another chromosome, typically chromosome 14 [17].

Children with Down syndrome exhibit a range of malformations in addition to cognitive impairments resulting from the presence of extra genetic material from chromosome 21 [18,19]. Although the phenotype varies, common characteristics that can lead experts to suspect Down syndrome includes reduced muscular tone (hypotonia), a brachycephalic head shape, a flat nasal bridge, epicanthal folds, the presence of Brushfield spots in the iris, a small mouth, small ears, excess skin at the back of the neck, upward-slanting palpebral fissures, a short fifth finger, a single transverse palmar crease, clinodactyly (abnormal curvature of the fifth finger), and wide spacing between the first and second toes, often accompanied by a deep groove between them [20,21]. The neurodevelopmental and cognitive profiles observed in individuals with Down syndrome are characterized by significant diversity, presenting unique challenges and opportunities for diagnosis, management, and support [22]. Comorbidities associated with Down syndrome further contribute to the complexity of providing comprehensive care to this population [23,24,25]. Cognitive impairment in individuals with Down syndrome can range from mild (with an IQ between 50 and 70) to moderate (with an IQ between 35 and 50), and occasionally, it can be severe (with an IQ between 20 and 35) [26,27]. Additionally, individuals with Down syndrome face a significant risk of experiencing hearing loss (75%), obstructive sleep apnea (50% to 79%), otitis media (50% to 70%), eye-related issues (60%) including cataracts (15%) and severe refractive errors (50%), congenital heart defects (50%), neurological dysfunction (ranging from 1% to 13%), gastrointestinal atresias (12%), hip dislocation (6%), and thyroid disorders (ranging from 4% to 18%) (Table 1) [28].

Artificial intelligence (AI) and machine learning (ML) have emerged as powerful tools with the potential to revolutionize various fields, including healthcare [29,30,31]. ML, as a subset of AI, focuses on enabling computers to learn from data and improve their performance on specific tasks without explicit programming [32]. While AI is a broader concept, ML plays a crucial role in the implementation of intelligent systems [33,34,35]. In recent years, AI and ML have gained significant attention in healthcare due to their potential to enhance diagnosis, prediction, and treatment planning for various conditions, including DS [36]. These technologies can analyze complex medical data, identify patterns and trends, and provide valuable insights for healthcare professionals and families affected by DS [36]. ML holds promise in the field of Down syndrome by facilitating early diagnosis, predicting associated medical conditions, and enhancing educational interventions [37]. By leveraging ML algorithms to analyze large datasets of genetic and clinical information, researchers and healthcare professionals can gain valuable insights that contribute to personalized care and improved outcomes for individuals with Down syndrome [36]. Given the diverse neurodevelopmental and cognitive profiles in individuals with Down syndrome and the complexities posed by associated comorbidities, this review aims to comprehensively analyze the existing literature. It specifically focuses on neurodevelopmental and cognitive features, comorbidities, and current approaches to diagnosis and management in Down syndrome. Additionally, it explores the potential role of ML and AI in enhancing Down syndrome care, emphasizing the need for careful evaluation and further research. By synthesizing the available information, this review aims to inform and guide healthcare practitioners in their efforts to provide effective and individualized care to individuals with Down syndrome.

## 2. Diagnostics

The prospective for the growth and socialization of Down syndrome affected individual has now been realized and improved with early intervention techniques, thereby timely support for DS affected children is extensively implemented [38,39,40]. With the introduction of first trimester screening, the options of diagnostics for Down syndrome have improved significantly. In addition to maternal age, the assessment includes nuchal translucency ultrasonography, along with the measurement of maternal serum human chorionic gonadotropin and plasma protein A in relation to the pregnancy [41,42,43]. The second-trimester screening incorporates the maternal age-related risk and involves measuring maternal serum hCG, unconjugated estriol, α-fetoprotein (AFP), and inhibin levels [44,45,46]. The first-trimester screening achieves a detection rate for Down syndrome ranging from 82% to 87%, while the second-trimester screening achieves an 80% detection rate. When both the first and second-trimester screenings are combined, often referred to as integrated screening, the detection rate increases to approximately 95% [47,48,49]. Early diagnosis, intervention, and ongoing support are crucial for individuals with Down syndrome to reach their full potential and lead fulfilling lives [50]. Early childhood intervention programs, involving a multidisciplinary approach, provide comprehensive support in areas such as speech, motor skills, cognition, and social-emotional development [50,51]. Individualized education plans (IEPs) tailor educational goals and accommodations to each child’s unique needs, promoting inclusive learning and skill development [9,50]. Medical management, including regular check-ups and proactive care for associated health conditions, ensures optimal health outcomes [52,53,54]. By emphasizing the importance of early interventions and support strategies, we highlight the need to empower individuals with Down syndrome and promote their development across multiple domains [9,50].

### 2.1. Prenatal Diagnostics

Parental awareness plays a crucial role in the context of Down syndrome, as it is essential for parents to possess a comprehensive understanding of the potential conditions associated with Down syndrome [55,56]. Such awareness can significantly contribute to the accurate diagnosis and appropriate treatment of this disorder [57,58]. The introduction of cell-free prenatal screening and the parallel sequencing of maternal plasma cell-free DNA (cfDNA) has brought about a profound transformation in the standard approach to prenatal Down syndrome diagnosis [47]. The utilization of non-invasive prenatal screening has the potential to reduce the need for invasive tests such as amniocentesis or chorionic villus sampling [59]. Furthermore, soft markers, including the absence or small size of the nasal bone, increased nuchal fold thickness, and enlarged ventricles, can be detected through ultrasound examinations performed between the 14th and 24th weeks of gestation [60,61]. An elevated fetal nuchal translucency measurement is indeed associated with an increased risk of Down syndrome. Increased fetal detection of Down syndrome offers important benefits despite the limited need for fetal or neonatal intervention in most cases [62]. Early detection enables comprehensive prenatal counseling, facilitating informed decision-making for expectant parents and access to specialized care and support. It respects individual autonomy, allowing families to make choices aligned with their values [63].

Moreover, increased detection contributes to research and advancements in prenatal care and treatments, driving improved outcomes for individuals with Down syndrome [50,62,63]. By accumulating data and insights, it enables the development of innovative interventions, early interventions, and support strategies. Therefore, advocating for increased fetal detection is crucial, as it empowers parents, facilitates specialized care, respects personal choices, and fuels research advancements [63]. In addition to these advancements, various methods are employed for prenatal diagnosis, with traditional cytogenic analysis remaining widely used in many countries. Nevertheless, some rapid molecular assays, such as fluorescent in situ hybridization (FISH), quantitative fluorescence PCR (QF-PCR), and multiplex probe ligation assay (MLPA), are also utilized for prenatal diagnosis [7]. Prenatal diagnosis provides valuable information about the chromosomal abnormality, but it does not directly inform us about the specific cognitive and neurodevelopmental traits that individuals with Down syndrome will exhibit [1]. Understanding this variability requires comprehensive research that explores cognitive profiles, strengths, and challenges in individuals with Down syndrome, considering environmental influences and personalized experiences [22,64]. It is crucial to acknowledge that while prenatal diagnosis provides valuable information about the chromosomal abnormality, it does not directly inform us about the wide variability in neurodevelopmental and cognitive characteristics that will be unique to each person with Down syndrome [65]. Indeed, the neurodevelopmental and cognitive profiles in individuals with Down syndrome exhibit significant diversity [66,67]. While the presence of an extra copy of chromosome 21 contributes to shared characteristics, such as intellectual disability and certain physical features, the specific cognitive abilities, strengths, and challenges can vary widely among individuals [68]. Factors such as genetic variations and individual differences contribute to this variability. In order to provide a comprehensive understanding of Down syndrome, it is crucial to consider beyond prenatal diagnosis [69]. Additional assessments, evaluations, and ongoing monitoring are necessary to capture the individual’s specific cognitive and neurodevelopmental traits. This includes evaluating cognitive abilities, language skills, motor development, adaptive functioning, and social-emotional aspects [69]. It emphasizes the need for personalized and individualized interventions that address the unique strengths, challenges, and needs of each person [65]. By considering the wide range of cognitive and neurodevelopmental profiles, practitioners can provide more effective and tailored support for individuals with Down syndrome [65,66]. Many countries have chosen to incorporate prenatal diagnosis into their healthcare systems, offering prospective parents an opportunity to make informed choices aligned with their personal values [70]. This encompasses decisions regarding whether to proceed with a pregnancy or consider termination of pregnancy (TOP). The integration of prenatal diagnosis respects individual autonomy by empowering families to navigate complex decisions in accordance with their unique values and beliefs. In recognizing the diversity of international practices, it is important to emphasize that the availability of prenatal diagnosis is not universally linked to the sole option of termination. Rather, it serves as a means to provide comprehensive information, fostering an environment where families can make decisions that align with their individual circumstances and ethical considerations [71,72].

### 2.2. Artificial Intelligence (AI)-Based Diagnosis

Medical lab tests, investigation of medical history, and genetic testing are all commonly used methods to diagnose Down syndrome. To help with the diagnosing process, artificial intelligence (AI) and machine learning (ML) approaches can be quite useful [30,35]. A variety of clinical data can be analyzed using AI and ML algorithms, which can be trained to identify patterns that might be symptomatic of Down syndrome. Incorporating ML techniques into Down syndrome detection holds significant potential for enhancing accuracy, efficiency, and accessibility [64,68,73]. The integration of machine learning (ML) into cell-free prenatal screening and maternal plasma cell-free DNA sequencing for Down syndrome diagnosis will present a transformative paradigm with significant motivations and potential enhancements. Early detection may be improved, and the potential for reduced false positives addresses concerns related to unnecessary interventions. ML’s adaptive nature ensures continuous improvement, contributing to the evolution of more precise and reliable prenatal Down syndrome predictions. ML algorithms enable the analysis of large datasets encompassing clinical and genetic information, potentially identifying subtle markers and patterns that improve detection accuracy beyond traditional methods [74]. Integrating multiple data sources, including maternal age, biochemical markers, and ultrasound measurements, ML-based predictive models can yield more sophisticated risk assessments and enable precise counseling for expectant parents [35]. ML methods offer broader accessibility and cost-effectiveness compared to invasive procedures like amniocentesis or chorionic villus sampling, as they primarily rely on non-invasive data sources such as maternal blood samples and medical records. Furthermore, ML techniques can be automated and scaled, facilitating widespread implementation and reducing the economic burden associated with DS screening [35,74]. While current diagnostic methods for Down syndrome exhibit high accuracy rates, incorporating ML methods can provide additional advantages in terms of improved accuracy, risk assessment, counseling, and broader accessibility. By leveraging ML algorithms to analyze comprehensive datasets, healthcare providers can enhance DS detection and deliver more personalized care [35]. These motivations and benefits of ML methods in Down syndrome detection will be further emphasized in the revised manuscript, supporting the advocacy for their integration. ML and AI can help with the diagnosis in the following ways:

#### 2.2.1. Facial Recognition

AI programs can be trained to identify facial characteristics that are commonly linked to Down syndrome [75]. ML models can recognize distinct features like an upward slope in the eyes, a flattened face profile, and a tiny nose by looking at facial images. These algorithms may precisely identify these features, assisting in diagnosis of Down syndrome [76].

#### 2.2.2. Genetic Screening

AI and ML can help with the analysis of genetic algorithm data to identify the early risk of Down syndrome [77]. Medical experts may input a person’s genetic sequence into an ML model, which can then compare it with a very large dataset of genetic profiles known to be associated with Down syndrome [36]. The system can assess the likelihood of Down syndrome and accurately identify biological markers.

#### 2.2.3. Analysis of Medical Data

AI algorithms can process patient medical records [78] to find patterns and links with Down syndrome. This analysis includes historical test results, developmental milestones, and symptoms. A huge collection of patient information can be used to train machine learning models to spot patterns or warning signs that are typical of the ailment [79]. It can thus aid medical professionals in developing more precise and effective diagnosis [80].

#### 2.2.4. Support for Prenatal Diagnosis

AI and ML can also help with Down syndrome prenatal diagnosis [49]. Artificial intelligence (AI) systems can spot possible indicators of Down syndrome in a growing fetus by examining ultrasound images [81] or blood test data. Because of the early detection, parents and medical professionals can better anticipate and support the child’s requirements.

#### 2.2.5. Decision Support Systems for Healthcare

By making timely and accurate recommendations based on patient data, AI and ML can serve as decision support tools for healthcare professionals [82]. ML models can predict the risk of Down syndrome through incorporating clinical and genomic data analysis, enabling healthcare practitioners to make well-informed decisions about additional diagnostic procedures or specialist referrals [83]. It is significant to remember that a medical practitioner should always validate the final diagnosis [84]. The purpose of AI and ML in the diagnosis of Down syndrome is to support medical practitioners by offering insightful information and improving the precision and effectiveness of the diagnostic procedure.

## 3. Cognitive Challenges in Down Syndrome

Cognitive functioning is the collective term for a variety of mental processes, such as retention, acquisition, reasoning, problem-solving, adaptability, and attention. Cognitive functioning, which ranges from profound to borderline intellectual capacity, is a hallmark of Down syndrome (DS) [8,85,86,87]. Most Down syndrome sufferers have moderate to severe intellectual disabilities. Cognitive growth goes on all the way through childhood, adolescence, and the first few years of adulthood. The loss of skills that are commonly associated with dementia gradually follows this. When compared to visual information, people with Down syndrome consistently have trouble understanding verbal information. Learning, memory, and language problems that cause mild to severe intellectual disability are characteristics of Down’s syndrome [85,86,88,89].

### 3.1. Speech, Mental Abilities, and Memory Retention

The cognitive profiles of those with the disease differ, with maintained visuospatial short-term memory, associative learning, implicit long-term memory, poor morphosyntax, verbal short-term memory, and explicit memory. Individuals with Down syndrome are better at pictorial tasks equated to verbal short-term memory tasks [8,90]. Although infants show less vocal response and environmental alertness than older children and adults, early language milestones are often met within an age-expected range. It has been shown that youngsters acquire their first words later than anticipated [85,86]. At the outset, it is usually recognized as a characteristic to have a small vocabulary, thoughtful communication, and pragmatism in language. The usage of multi-word sentences is delayed as linguistic demands rise, and strange communication patterns emerge. Persistent language problems are noticed after a child is five. The language profiles of school-aged children reveal a noteworthy lag in the progression of expressive language when compared to receptive language. This discrepancy is most pronounced in the domains of expressive syntax and phonological processing, where the most substantial delays are observed [87,91,92]. Syntactic insufficiency is mainly evident in late infancy and the start of puberty. Adults have less phonological processing, morphosyntax, and articulation issues with language, but their semantic, pragmatic, and communicative goals remain largely unaltered. Learning, memory, and other cognitive processes can all suffer from impaired language comprehension processing [86,88,93,94].

### 3.2. Processing Speed, Inhibition, and Attention

The executive functions (EFs), which control behavior and cognition, include things like attention, inhibition, and processing speed. Higher level executive function includes skills like strategic planning, impulse control, systematic search, flexibility of thought and action, and the ability to blend what one wants with what they can do [94]. Teenagers with Down syndrome perform worse on tests of attention, perceptual quickness, response time, and motor control when compared to peers with similar mental ability. These limitations persist as individuals age, making it more challenging to allocate tasks, retain attention, and respond reliably to situations [95,96]. Poor response inhibition is evident across the whole developmental lifespan, with vocally mediated inhibition tasks being more difficult and having poor inhibition of irrelevant information. Response time assessments yield contradictory results, with faster reaction times compatible with intellectual functioning but slower than those with mental age matching individuals who possess intellectual disability [87,93].

### 3.3. Short-Term Auditory Memory

The visuospatial working memory system is more developed than the auditory working memory system, and verbal working memory deficits go beyond those seen in those who have difficulty hearing and speaking well [93,97]. Lack of engaged learning may contribute to diminished verbal memory retention in scholastic age adolescents and kids. The ability to recall information correlates with syntax interpretation in both modalities, illustrating the relationship between working memory and linguistic acquisition. When compared to verbal working memory, tasks requiring less information or when the visual and spatial components are assessed separately still have little impact on visual and spatial short-term memory [98,99]. Children with Down syndrome have trouble with problem-solving techniques, and as they become older, they take longer to complete planning activities, even when the results are similar to those of children whose mental ages are matched. Multitasking and time shifting are exceedingly challenging for children and persons with Down syndrome, especially when it comes to vocally mediated tasks [100]. People with Down syndrome commonly experience verbal comprehension, self-monitoring, and executive function deficits, in contrast to other genetic ID-related disorders. Additionally, they erroneously and more slowly assimilate information [93,101,102,103].

### 3.4. Organization, Spatial Cognition, and Self-Monitoring

Children with Down syndrome frequently experience difficulties with integrating new knowledge and problem-solving techniques, which delays down their developmental progress. As individuals age, scheduling tasks take longer to accomplish, but their efficiency is comparable to that of mental age matched controls [104]. For kids and people with Down syndrome, multitasking and setting changing are extremely difficult, especially when it comes to vocally mediated activities. Additionally, people with Down syndrome struggle with verbal comprehension and self-awareness, frequently failing to indicate when they have understood something [105,106]. Due to poor monitoring for intrusion mistakes and problems avoiding irrelevant information from interfering with cognitive processes, adults with Down syndrome still have trouble self-monitoring. The profile of visual-spatial ability in people with Down syndrome is uneven, with some parts matching average cognitive capacity and others falling short of projected developmental levels. Though cognitive function is deteriorating, visuospatial abilities are still mostly intact [86,107,108].

### 3.5. Learning and Long-Term Memory

Children with Down syndrome have distinct degrees of learning ability, with diminished short-term and long-term memory learning abilities [109,110]. They do better at combining rewards with objects and with observational learning, but exhibit trouble with instrumental learning [111,112]. They are more socially inclined and receptive to positive reinforcement, enhancing the success in socially oriented learning. Visual learning is more efficient than verbal learning, which shows that interpersonal abilities are robust. Problems in attention and a high demand for processing contribute to long-term memory problems in Down syndrome at the encoding and retrieval levels [113]. These deficits might be intrinsic in origin rather than just a symptom of a language processing disorder. These inadequacies persist throughout life, but they worsen with advancing years [103].

### 3.6. Associated Conditions and Disorders

People with Down syndrome are more likely to have a number of different health issues, such as Dementia, autism spectrum disorders, hormonal, glandular issues, sensory impairments, sleep disruption, seizures, and cardiac abnormalities [114,115]. Celiac disease, hypothyroidism, leukemia, congenital heart abnormalities, and diabetes are additional illnesses with increased occurrence in this group [85,86,116,117]. Many people with Down syndrome are born with congenital heart defects, such as atrioventricular septal defect or ventricular septal defect. These heart conditions may necessitate surgical intervention [114]. Hearing issues, including conductive or sensorineural hearing loss, are frequently observed in individuals with Down syndrome. Regular hearing assessments are crucial for early intervention [115]. Ocular problems like cataracts, strabismus (crossed eyes), and refractive errors are more common among those with Down syndrome [118]. Hypothyroidism, which is an underactive thyroid gland, is more prevalent in people with Down syndrome. Routine monitoring of thyroid function is of utmost importance [117].

## 4. Discussion

**Figure 1:**
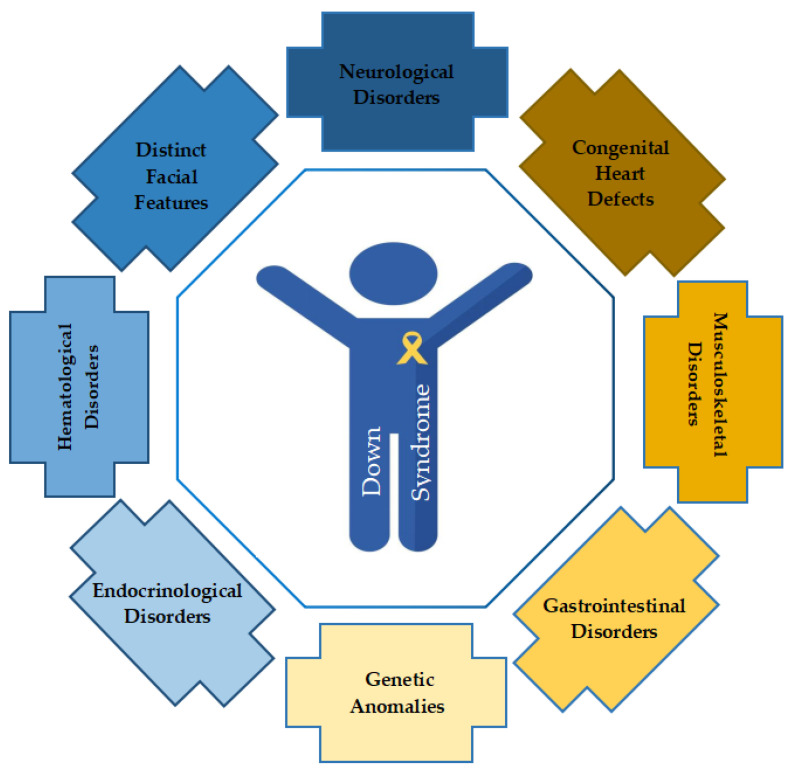
Down syndrome and neurocognitive profiles associated disorders.

Down syndrome, caused by a genetic anomaly (trisomy 21), manifests in characteristic physical features and cognitive delays [9]. Individuals often contend with a range of comorbidities, including heart defects, gastrointestinal issues, and increased susceptibility to infections. These additional health concerns necessitate comprehensive medical care and early interventions to address associated challenges and optimize overall well-being [8,9]. Numerous co-morbidities (Figure 1) identified such as congenital heart defects, celiac disease, gastrointestinal defects, seizures, thyroid disease, hematological disorders, autism, and emotional and behavioral disorder (EBD) are known to affect the quality of life in children with Down syndrome [8,119]. Table 2 presents the various specific disorders/diseases as subcategories of these co-morbidities. Individuals with Down syndrome are also predisposed to sleep disorder breathing (SDB) which includes central sleep apnea (CSA), hypoxemia disorder, hypoventilation disorder, and obstructive sleep apnea (OSA) [120,121]. Central airway anatomical features such as small oropharynx, mid-facial hypoplasia, narrow nasopharynx, and macroglossia contribute DS towards increased susceptibility for SDB [122,123]. Many previous studies have reported SDB high prevalence associated with Down syndrome condition compared to the general population [124,125,126]. Douglas Bush et al., in a retrospective large cohort study (n = 1242), identified high incidence (28%) of pulmonary hypertension with associated co-morbidities such as OSA, chronic hypoxia, recurrent pneumonia, and aspiration in patients with DS [127]. Early management of respiratory disorders contributes towards improved condition and reduced susceptibility of pulmonary hypertension in individuals with Down syndrome. Reports based on co-morbidity epilepsy (seizure disorder) showed increased prevalence in individuals (8.1–26%) with Down syndrome compared to general population (1.5–5%) [128]. Major biological and metabolic factors present in Down syndrome patients contributing to increased seizures include dyskinesia of dendrites, frontal/temporal lobe hypoplasia, abnormal neuronal lamination, glutamatergic receptor GluR5 alteration, and congenital heart disease [129,130]. Following seizures, there is a profound connection with other associated co-morbidity i.e., dementia in Down syndrome patients. Hithersay et al., in a prospective longitudinal study, found individuals in older age and late-onset of epilepsy were associated with increased risk of developing dementia in Down syndrome cases [131]. Another cross-sectional study by Bayen et al. determined high prevalence of dementia in DS adults above 65 years with marked risk of developing Alzheimer disease (AD) [132]. Further, a neuroimaging study by Pujol et al. based on adults with DS showed significant volume reduction in hippocampus and substantia innominata of brain anatomy specifically linked to cognitive impairment and dementia progression [133]. Early diagnosis of dementia and AD in DS individuals is not possible due to pre-existing behavioral and intellectual disorders. Recently, a study by Dekker et al. based on behavioral and psychological symptoms of dementia in Down syndrome (BPSD-DS) scale identified behavioral changes such as anxiety, agitation, depression, sleep disturbance, and apathy had significantly high scores in DS+AD (Down syndrome with AD) compared to DS+Q (Down syndrome with questionable dementia) and without dementia individual study groups [62,134]. Based on other behavioral studies, individuals with DS presented symptoms such as sleep disturbance, anxiety, depression, and apathy as alarming signs for developing AD [135,136,137]. Other neurodevelopment disorders associated with DS include autism spectrum disorder (ASD) and attention deficit hyperactivity disorder (ADHD), as investigated in recent population based cohort study showing 42% ASD and 34% ADHD prevalence in DS individuals [138]. Pre-existing intellectual disability associated with Down syndrome might be the facilitating factor for the characteristic heterogeneity in ASD symptoms. Congenital heart defects (CHDs) are one of the profound co-morbidities associated with DS as the prevalent cause of infant mortality [139,140,141,142,143]. Baban et al. investigated the frequency of Down syndrome infants (N = 859) for CHD subtypes based on a single center study, reporting a high proportion with CHDs (72.2%) and 4.7% with atypical CHDs [144]. Following research for DS-CHD (DS associated with CHD) trend in infants present less frequency mainly due to selective abortion of fetus or diagnostic improvement for managing antenatal CHD [113,145]. Patients with DS are reported to present two common types of cardiac defects such as atrioventricular septal defect (45%) and ventricular septal defects (20–30%), respectively [71,146]. The prevalence of different co-morbidities associated with Down syndrome varies across the geographical population [147,148]. Further, the majority of co-morbidities generally requires clinical and psychiatric management with not much effect on mortality, except CHD and epilepsy. Future management of patients with DS thus requires proper understanding of the co-morbidities associated for providing appropriate help they need [147,148].

ML algorithms have been employed to analyze large datasets of genetic and clinical information to gain insights into Down syndrome and improve patient care. Down syndrome is a genetic disorder caused by the presence of an extra copy of chromosome 21, leading to cognitive and developmental delays [8]. ML techniques have been used to identify biological markers and patterns associated with Down syndrome. By analyzing genomic data from individuals with Down syndrome and comparing it with data from typically developing individuals, ML algorithms can identify specific genetic variations or expression patterns that are characteristic of the condition [74,149]. These studies present pioneering applications of artificial intelligence (AI) and machine learning (ML) in Down syndrome (DS) research. Another study employs ML to scrutinize clinical records of 106 DS subjects, successfully identifying key features associated with intellectual disability (ID). The models, including random forest and gradient boosting, showcase high accuracy, spotlighting variables linked to cognitive impairment, encompassing hearing, gastrointestinal health, thyroid function, immune system, and vitamin B12 levels [74]. In a second study, addressing executive function decline in adults with DS, data-driven techniques pinpoint constructive praxis, verbal and immediate memory, planning, and written verbal comprehension as crucial predictors for inhibition capacity in 188 adults, providing insights for tailored interventions [149]. This can aid in early diagnosis, genetic counseling, and personalized treatment strategies. Furthermore, ML algorithms can assist in the development of predictive models for assessing the risk of certain medical conditions commonly associated with Down syndrome [73,150]. Another study addresses the frequent occurrence of obstructive sleep apnea (OSA) in individuals with Down syndrome. Using a Logic Learning Machine, the study develops a predictive tool with a cross-validated negative predictive value of 73% for mild OSA and 90% for moderate or severe OSA. This cost-effective model includes survey responses, medication history, anthropometric measurements, vital signs, age, and physical examination findings, offering potential improvements to sleep-related healthcare [73]. ML also plays a role in improving educational interventions and therapies for individuals with Down syndrome [74]. By analyzing data from educational programs, ML algorithms can identify effective teaching strategies, personalize learning approaches, and provide recommendations for individualized educational plans [74]. Additionally, ML-based technologies, such as speech and language processing algorithms, can assist in speech therapy and communication interventions for individuals with Down syndrome [68]. This study provides an in-depth analysis of AI-driven solutions that enhance communication and education for disabled children, concluding with considerations for future developments and ethical concerns associated with these technologies [68]. Together, these studies showcase the multifaceted applications of AI and ML in advancing understanding, diagnosis, and care for individuals with DS. It is important to note that the application of ML in Down syndrome research and healthcare requires careful consideration of ethical and privacy considerations. Ensuring the responsible use of data, protecting privacy, and addressing potential biases in algorithms are crucial aspects that need to be addressed to fully harness the potential of ML in improving the lives of individuals with Down syndrome [151]. These studies collectively illuminate the transformative potential of artificial intelligence (AI) and machine learning (ML) in diverse fields. From enhancing Down syndrome diagnosis by pinpointing key cognitive indicators and predicting inhibitory capacity to predicting sleep apnea risk and advancing assistive technologies for children with special needs, the application of AI and ML showcases promising avenues for precision, efficiency, and innovative solutions. Additionally, these studies also underscore the indispensable role of AI in addressing data challenges across industries, offering valuable insights and strategies for effective implementation. Furthermore, these findings collectively underscore the significant impact of AI and ML in reshaping research, diagnosis, and intervention strategies across various domains.

The present review extends prior research by providing a comprehensive exploration of Down syndrome’s multifaceted dimensions. By synthesizing insights into neurodevelopmental aspects, associated comorbidities, and the integration of artificial intelligence (AI), this study offers a significant extension of existing knowledge. The implication of our review lies in its potential to steer future research, emphasizing the need for sophisticated knowledge and technological advancements in AI for a more precise understanding of Down syndrome. The ultimate goal is to leverage AI’s potential to enhance diagnostic accuracy, intervention strategies, and therapeutic advancements. However, challenges persist, notably in data quality, interpretability, and ethical considerations. While highlighting AI’s transformative merits and potential clinical applications, we acknowledge the limitations and call for future research focusing on refining methodologies and ethical frameworks to maximize AI’s benefits in Down syndrome research and care pathways. These technologies can improve screening, diagnosis, and personalized interventions, ultimately benefiting individuals with Down syndrome. However, the responsible and ethical use of ML and AI in Down syndrome care requires ongoing research, validation, and careful consideration of privacy and fairness concerns. This line of research holds immense significance in reshaping our approach to Down syndrome, paving the way for impactful innovations and improving the lives of individuals affected by this condition. By harnessing the power of ML and AI responsibly, practitioners can improve outcomes and provide better care for individuals with Down syndrome. The continued collaboration between researchers, healthcare practitioners, policymakers, and the Down syndrome community will drive further progress towards enhancing the lives and prospects of individuals with Down syndrome. Future studies should delve into these complexities to inform tailored interventions and support systems that promote positive outcomes for individuals with DS and their families.

## 5. Conclusions

In conclusion, this comprehensive review has highlighted the neurodevelopmental and cognitive characteristics observed in individuals with Down syndrome. The findings underscore the diverse nature of this population, emphasizing the importance of tailored interventions and support techniques. Moreover, emerging research has demonstrated the potential of ML and AI algorithms in accurately identifying individuals at risk of Down syndrome, aiding healthcare practitioners in early detection and intervention. While individuals with Down syndrome may face cognitive challenges, it is crucial to recognize and nurture their unique skills and qualities. By promoting inclusive environments and providing customized support, we can empower individuals with Down syndrome to reach their full potential and enhance their overall quality of life. Looking towards the future, ongoing advancements in research and therapeutic interventions offer promising prospects for individuals with Down syndrome. By further understanding the underlying mechanisms and exploring innovative approaches, we can develop targeted interventions that address specific cognitive and behavioral aspects. This, in turn, will enable individuals with Down syndrome to attain greater levels of autonomy and well-being. Societal progress and awareness play pivotal roles in fostering an inclusive and supportive environment for individuals with Down syndrome. By prioritizing the cultivation of an inclusive society, we can create opportunities for meaningful participation, education, and employment for individuals with Down syndrome. This will contribute to their social integration and overall quality of life.

## Figures and Tables

**Table 1:** Down syndrome associated complications.

S. No.	Down Syndrome Associated Complications	Occurrence
1.	Cataracts	15%
2.	Congenital heart ailments	40–50%
3.	Dental eruption (Delayed)	23%
4.	Gastrointestinal atresias	12%
5.	Hearing issues	75%
6.	Hip dislocation	6%
7.	Neurological Impairment	1–13%
8.	Otitis media	50–70%
9.	Refractive errors	50%
10.	Sleep apnea (Obstructive)	50–75%
11.	Thyroid disorders	4–18%
12.	Vision impairments	60%

**Table 2:** Co-morbidities and corresponding disorders/diseases.

Co-Morbidity	Disorder/Disease
Neurological Disorders	Alzheimer disease Dementia Excessive flexibility Intellectual disability Learning disability Lennox–Gastaut syndrome Less concentration Seizures
Congenital Heart Defects	AVS defect Isolate PDA SA defect Tetralogy of Fallot VS defect
Musculoskeletal Disorders	Broad small hands Decreased bone mass Growth retardation Hypotonia Short fingers Short height Vitamin D deficiency Small feet
Gastrointestinal Disorders	Celiac disease Chronic constipation Duodenal atresia Gastroesophagal reflux Hirschsprung disease Imperforate anus Intermittent diarrhea Intestinal obstruction
Possible Genetic Anomalies	Mosaicism Translocation Trisomy 21
Endocrinological Disorders	Ambiguous genitalia Cryptorchidism Delayed puberty Micropenis Hyperthyroidism Hypothyroidism
Hematological Disorders	Leukemia Myelopoiesis Neutrophilia Polycythemia Thrombocytopenia
Distinct Facial Features	Flattened face and nose Palmer/Siamese crease Palpebral fissures Protruding tongue Short neck Slanting eyes Small head, mouth, and ears

## Data Availability

Data are contained within the article.
